# Developmental Trajectories of Internalizing and Externalizing Symptoms in Youth and Associated Gender Differences: A Directed Network Perspective

**DOI:** 10.1007/s10802-023-01106-4

**Published:** 2023-08-07

**Authors:** Kevin Liu, Ryan C. Thompson, Jessica Watson, Alexandra L. Montena, Stacie L. Warren

**Affiliations:** 1 Department of Psychology, Palo Alto University, Palo Alto, CA USA; 2 Department of Psychology, School of Behavioral and Brain Sciences, The University of Texas at Dallas, TX Richardson, USA

**Keywords:** Developmental psychopathology, Psychopathology networks, Transdiagnostic, Graphical vector autoregressive model

## Abstract

Psychopathology in youth is highly prevalent and associated with psychopathology in adulthood. However, the developmental trajectories of psychopathology symptoms, including potential gender differences, are markedly underspecified. The present study employed a directed network approach to investigate longitudinal relationships and gender differences among eight transdiagnostic symptom domains across three years, in a homogenous age sample of youth participants (*n* = 6,414; mean baseline age = 10.0 years; 78.6% White; Adolescent Brain Cognitive Development study). Anxious/depressed problems and aggressive behaviors were central symptoms and most predictive of increases in other symptom clusters at later timepoints. Rule-breaking behaviors, aggressive behaviors, and withdrawn/depressed problems emerged as bridge symptoms between externalizing and internalizing problems. Results supported cascade models in which externalizing problems predicted future internalizing problems, but internalizing problems also significantly predicted future externalizing problems, which is contrary to cascade models. Network structure, symptom centrality, and patterns of bridge symptoms differed between female and male participants, suggesting gender differences in the developmental trajectories of youth psychopathology. Results provide new insights into symptom trajectories and associated gender differences that may provide promising pathways for understanding disorder (dis)continuity and co-occurrence. The central and bridge symptoms identified here may have important implications for screening and early intervention for youth psychopathology.

**Supplementary Information:**

The online version contains supplementary material available at 10.1007/s10802-023-01106-4.

## Developmental Trajectories of Internalizing and Externalizing Symptoms in Youth and Associated Gender Differences: A Longitudinal Network Perspective

Rates of psychopathology in youth are high, with an estimated U.S. lifetime prevalence > 20% of disorders with severe impairment and/or distress (Merikangas et al., 2010). A substantial proportion of adult psychopathology diagnoses have their origins in childhood and adolescence. For example, approximately two-thirds of lifetime depression cases among adults emerged in adolescence (Kessler et al., 2005), and nearly all anxiety disorders begin in childhood (Kessler et al., 2009). Additionally, antisocial personality disorder in adult men is associated with a diagnostic trajectory of attention-deficit/hyperactivity disorder (ADHD), oppositional defiant disorder, and other diagnoses in childhood (Beauchaine et al., 2017). Variability in psychopathology (dis)continuity between childhood and adulthood (Rutter et al., 2006; Shevlin et al., 2017) highlight a critical need to understand psychopathology emergence and progression across development. Given the heterogeneity of disorder presentation and remarkable disorder co-occurrence, including between internalizing and externalizing disorders (e.g., nearly 50% of children with ADHD experience co-occurring depression; Wilens et al., 2002), the application of categorical approaches (e.g., *Diagnostic and Statistical Manual of Mental Disorders*) may obscure developmental pathways of symptom trajectories. Understanding how symptoms may influence one another independent of diagnostic categories may provide insights that resolve disorder heterogeneity and co-occurrence. Here, we used a directed symptom-level network approach to characterize how internalizing and externalizing symptoms at baseline predict future symptoms within a homogenous age group of children.

Various methods have been used to study the development of psychopathology in youth, including epidemiological studies. For example, studies have examined the rates of different psychiatric disorders across childhood and adolescence, as well as proposed a general psychopathology factor that measures commonality among various disorders during youth (Costello et al., 2011; Patalay et al., 2015). These studies have yielded intriguing findings, including an adolescence-limited increase in generalized anxiety disorder prevalence among girls. In contrast, other anxiety disorders, such as social anxiety disorder and specific phobia, demonstrate a consistent increase in prevalence across adolescence and into adulthood (Costello et al., 2011). Additionally, a general psychopathology latent factor explains shared variance across internalizing and externalizing disorders during childhood and adolescence, suggesting similar developmental pathways or risk factors between internalizing and externalizing domains (Patalay et al., 2015). Research investigating gender differences in the development of youth psychopathology has demonstrated that boys experience higher rates of conduct disorders and girls experience higher rates of depression and anxiety (Lahey et al., 2000; Zahn-Waxler et al., 2008). However, previous research examining the broad spectrum of psychiatric disorders in youth have largely relied on cross-sectional data, preventing firm conclusions about the temporal dynamics or developmental pathways of psychopathology. As conflicting symptom and disorder progression have been reported in prior literature (e.g., Costello et al., 2011; Shevlin et al., 2017), comprehensive, longitudinal approaches are critical for delineating the nature of internalizing and externalizing symptom trajectories.

The application of a directed network approach to understanding psychopathology has broad appeal as it tests causal relations among symptoms, delineating their developmental trajectories (Borsboom, 2008; Cramer et al., 2010). A network approach emphasizes mutually reinforcing relationships between symptoms across disorders, distinguishing itself from the latent factor approach to psychopathology in which disorders are thought to be the common cause through which presenting symptoms can be explained (Kendler, 2016). Though a detailed discussion comparing network and latent factor approaches is beyond the scope of this paper, it is notable that these approaches can be combined, such as with latent network modeling and residual network modeling (Epskamp et al., 2017). Still, a network perspective can yield unique insights into the development and treatment of psychopathology. For example, the centrality hypothesis argues that symptoms which demonstrate high centrality (i.e., more numerous and stronger inter-symptom causal connections) are the most influential symptoms in both the development and remission of disorders (Borsboom & Cramer, 2013; Cramer et al., 2010). Additionally, symptoms that have causal relationships with symptoms from different clusters, such as depression and anxiety, can be seen as “bridge symptoms” that provide a potential causal mechanism and explain disorder comorbidity (Cramer et al., 2010). For example, Robinaugh et al. (2014) found loneliness to be a bridge symptom between persistent complex bereavement disorder and depression symptoms. However, these conclusions were drawn from cross-sectional networks rather than directed networks derived from longitudinal data, limiting inferences about the causal direction and temporal dynamics of the observed relationships.

Longitudinal studies of youth psychopathology have used a developmental cascade perspective to investigate the mutual influence of internalizing problems (e.g., anxiety, depression, social withdrawal) and externalizing problems (e.g., peer aggression, rule-breaking) over time (Dearing et al., 2006; Masten et al., 2009). Studies have shown reciprocal associations across time between internalizing and externalizing problems and how co-morbid symptoms can arise in youth (Achenbach & Rescorla, 2000; Mesman et al., 2001). Proposed causal mechanisms of these reciprocal associations include externalizing problems pre-disposing children to social rejection or academic failure thus leading to depression, particularly in boys (Patterson & Capaldi, 1990). However, conflicting evidence exists in the dynamics of these developmental cascades, with some studies finding that earlier externalizing problems predicted fewer internalizing problems later on (Panayiotou & Humphrey, 2018) and other studies finding that prior externalizing problems positively predicted later internalizing problems (Masten et al., 2005; Moilanen et al., 2010). Importantly, gender differences have also been found in these temporal dynamics with findings that internalizing problems predicted future externalizing problems consistently for girls but not boys (D’urso & Symonds, 2022), though the cascade effect of externalizing problems positively predicting later internalizing problems appeared consistent for both male and female participants (D’urso & Symonds, 2022; van Lier & Koot, 2010). More recently, Speyer et al. (2022) used network approaches to investigate developmental cascades and found that ADHD symptoms were highly central to development of later socioemotional symptoms and that prosocial behaviors served as a potential bridge symptom between externalizing and internalizing difficulties. Black et al. (2022) also utilized a network approach and found complex within-person effects between internalizing symptoms and indicators of well-being with indicators such as thinking clearly, unhappiness, dealing with stress, and worry being most central in the network.

The present study seeks to broaden understanding of developmental cascades by testing longitudinal relationships among sum scores of eight transdiagnostic symptom clusters––anxious/depressed, withdrawn/depressed, somatic complaints, social problems, thought problems, attention problems, rule-breaking behavior, and aggressive behavior––in pre-adolescents and adolescents over three timepoints (i.e., baseline, 1-year follow-up, and 2-year follow-up) using graphical vector autoregression (GVAR). GVAR models identify temporal relationships between symptoms by estimating edges, which represent the unique causal effects of one symptom cluster on another (Epskamp, 2020). By examining centrality indices, GVAR models can identify central symptoms that are most predictive of, or predicted by, other symptoms. The present study also tested gender differences in the longitudinal relationships among symptom clusters to highlight possible differences in developmental trajectories of psychopathology. Based on past studies that have identified depressed mood, attention difficulties, and anxiety as being the most central to psychopathology development in youth (Funkhouser et al., 2021; McElroy et al., 2018a, b), we hypothesized that anxious/depressed, withdrawn/depressed, and attention problems would be the most central symptoms clusters, influencing changes in other symptom clusters at later timepoints. Additionally, we hypothesized that symptom clusters would group together such that internalizing and externalizing domains would exhibit higher within-group symptom associations than between-group symptom associations, though we anticipated depressive symptoms to be a bridge between internalizing and externalizing disorders based on previous research (McElroy et al., 2018a, b).

## Method

This study used data collected from pre-adolescents and adolescents at baseline, 1-year follow-up, and 2-year follow-up assessments from the Adolescent Brain Cognitive Development (ABCD) study (data release 3.0; NDAR-https://doi.org/10.15154/1520926). The ABCD study is an ongoing, longitudinal study within the United States that follows a nationally representative sample of 11,878 children aged 9–10 at baseline (see Garavan et al., 2018 for information on sampling strategies across 21 data collection study sites, school and participant recruitment procedures, and informed consent; see Auchter et al., 2018 for details on ABCD study Institutional Review Boards, Bioethics and Medical Oversight advisory group, and other advisory boards). To test temporal associations of directed symptom network structures in the development of psychopathology, we examined a subsample (*n* = 6,414) who completed the assessment procedure at all three timepoints (timepoint 1 mean age = 10.0 years [*SD* = 0.6]; timepoint 3 mean age = 12.0 years [*SD* = 0.6]; 78.6% White; 82.4% 4^th^ or 5^th^ Grade at timepoint 3). Demographic information of the subsample is presented in Table S1. Gender identity of participants for the purposes of the study was defined by parent-report at 2-year follow-up. There were no significant differences in racial identity (χ^2^(180) = 192, *p* = 0.256), ethnicity (χ^2^(4) = 6, *p* = 0.199), and combined family income (χ^2^(180) = 192, *p* = 0.256) between participants identifying as male and female.

### Measures

Dimensional assessment of anxious/depressed, withdrawn/depressed, somatic complaints, social problems, thought problems, attention problems, rule-breaking behavior, and aggressive behaviors syndrome scales, representing symptom clusters, were assessed with the Child Behavior Checklist Parent’s Report Form (CBCL; Achenbach, 2001). On the CBCL, anxious/depressed, withdrawn/depressed, and somatic complaints are grouped as internalizing problems, while rule-breaking and aggressive behaviors are grouped as externalizing problems. *T*-scores normed by sex, age, and ethnicity were used for the present study analyses. *T*-scores had a lower bound of 50, representing 50th percentile or below, and an upper bound of 100, representing above 99th percentile. CBCL syndrome scales demonstrated one week test–retest reliability ranging from 0.80 to 0.94 (Achenbach, 2001). These syndrome scales have demonstrated concurrent validity with clinical diagnoses of anxiety disorders, mood disorders, attention-deficit/hyperactivity disorder, oppositional-defiant disorder, and conduct disorder (Ebesutani et al., 2010; Eiraldi et al., 2000; Kasius et al., 1997; Seligman et al., 2004).

### Statistical Analysis

GVAR models for panel data (Panel GVAR) were used to examine longitudinal relationships between scores on CBCL syndrome scales, representing symptom clusters, across baseline, 1-year follow-up, and 2-year follow-up time points. Panel GVAR models illustrate how CBCL syndrome scale scores influence each other and themselves across time at the within-person level while controlling for between-person differences in these scales. Additionally, Panel GVAR constrains the effects of the syndrome scale scores so that they are stable across the three timepoints in order to assess for stable effects across timepoints rather than deviations between timepoints.

Panel GVAR models were computed using full information maximum likelihood estimation. First, the full model was estimated, then a model search procedure was used to maximize the Bayesian information criterion (BIC) by pruning edges that were not statistically significant at the *p* < 0.05 level and adding edges that were significant at the *p* < 0.05 level. The comparative fit index (CFI; Bentler, 1990), Tucker-Lewis index (TLI; Tucker & Lewis, 1973), and root mean square error of approximation (RMSEA; Steiger & Lind, 1980) were calculated to assess model fit. Centrality indices were then calculated for each syndrome scale score in the final model. Instrength centrality represents the degree to which syndrome scales are predicted by scores on other scales at the previous timepoint, while outstrength centrality represents to what degree syndrome scales predict scores on other scales at the next timepoint.

Similarly, bridge in-degree centrality represents the degree to which each syndrome scale in one community (i.e., internalizing or externalizing) is predicted by syndrome scales in the other community at the previous timepoint, while bridge out-degree centrality represents to what degree syndrome scales in one community predict scores on scales in the other community at the next timepoint. Gender differences in network structures were tested by estimating the Panel GVAR model as a multi-group model, constraining parameters to be equal across gender groups, and assessing significance of change in model fit. Of note, the 13 participants who identified as transgender/other or for whom gender identity was not known were not included in the multi-group model separating male and female groups, though they were included in the full Panel GVAR model consisting of all 6,414 participants. More details regarding specifics of Panel GVAR models can be found in Epskamp (2020). Given concerns regarding the stability of network models (Forbes et al., 2019), the robustness of the estimated networks was tested by applying the same model search procedure for Panel GVAR models of 1,000 non-parametrically bootstrapped samples and calculating how often each edge was included in the optimal model, with 50% inclusion probability being considered robust (see Betz et al., 2020). Stability of centrality estimates was determined using case-dropping subset bootstrap, in which 20% of the sample was randomly dropped and the model was re-estimated across 1,000 iterations, and calculating 95% bootstrapped confidence intervals (see Epskamp et al., 2018a, b). All analyses involving Panel GVAR models were conducted using the *R* package “psychonetrics,” version 0.8.1. R code for study analyses presented here is available on the Open Science Framework (https://osf.io/fcuhm/?view_only=54cc8a191eed4da9a5a576bc35ef8c5b).

## Results

### Descriptive Statistics

Descriptive statistics for each of the CBCL subscales are described in Table S2. Each of the CBCL subscales were positively skewed and leptokurtic, as expected given the CBCL subscales’ restricted lower bound.

### Network Structure and Symptom Centrality

**Fig. 1:**
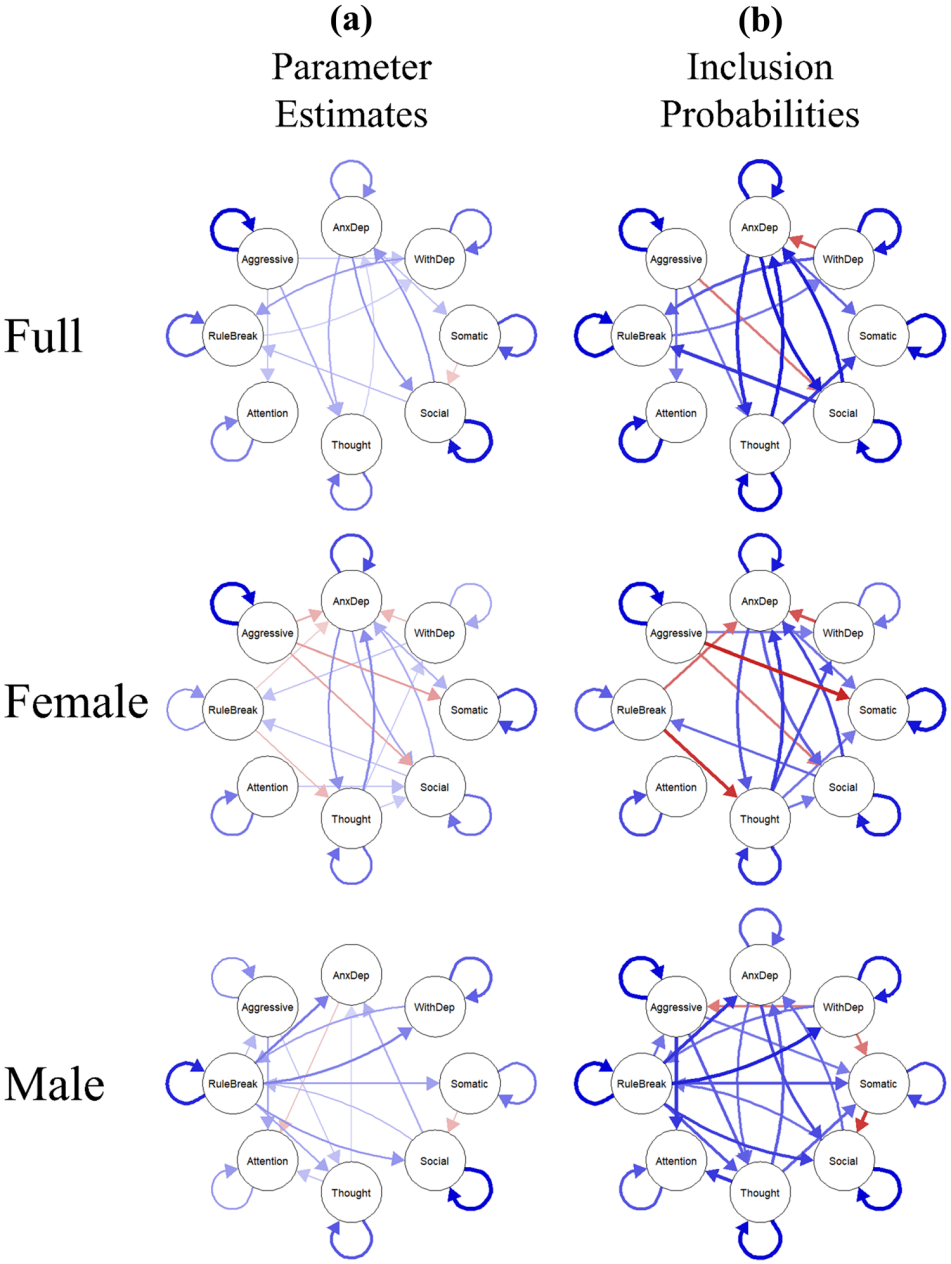
Temporal Network Result. Note. **a** all significant (p < 0.05) predictive and autoregressive effects between symptom domains with thicker lines indicating stronger effects, blue indicating a positive effect, and red indicating a negative effect. **b** All robust effects (inclusion probability > 50%) from the bootstrapped robustness analysis for the temporal network with thicker lines indicating greater inclusion probability, blue lines indicating positive effects, and red lines indicating negative effects. The symptom domains are: Anxious/Depressed Problems (AnxDep), Withdrawn/Depressed Problems (WithDep), Somatic Complaints (Somatic), Social Problems (Social), Thought Problems (Thought), Attention Problems (Attention), Rule-Breaking Behavior (RuleBreak), and Aggressive Behavior (Aggressive)

**Fig. 2:**
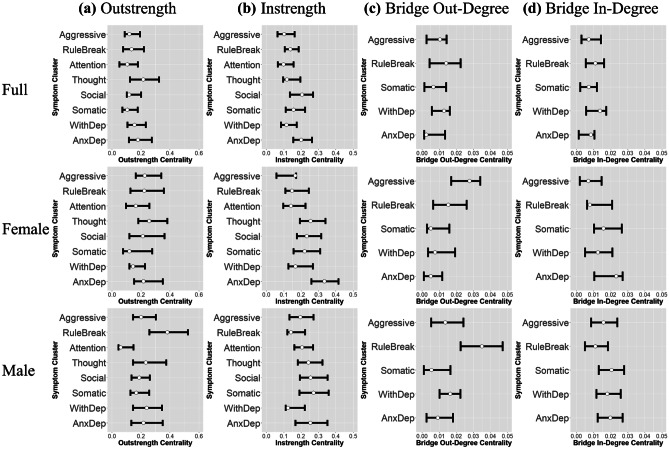
Centrality Estimates for Temporal Network. Note. Graphical representation of **a** outstrength, **b** instrength, **c** bridge out-degree, and **d** bridge in-degree centrality estimates of symptom domains from temporal network of the full sample, the female group, and the male group. Higher numerical values on the x-axis represent greater centrality, specifically greater number of edges and higher magnitude of edge weights for each symptom domain. Dot represents centrality estimate from temporal network estimated from the original sample, while bars indicate 95% bootstrapped confidence interval based on 1,000 iterations of case-drop subset bootstrap. The symptom domains are: Anxious/Depressed Problems (Anxious/Depressed), Withdrawn/Depressed Problems (Withdrawn/Depressed), Somatic Complaints (Somatic), Social Problems (Social), Thought Problems (Thought), Attention Problems (Attention), Rule-Breaking Behavior (Rule Breaking), and Aggressive Behavior (Aggressive)

The temporal network (i.e., the network of temporal effects of the eight CBCL syndrome scales on themselves and each other across the three timepoints) was estimated (see Fig. 1a). All significant edges included in the final model are shown in Table 1. The final model demonstrated good fit (CFI = 0.991, TLI = 0.989, RMSEA = 0.0269). All autoregressive effects, representing predictive associations between a CBCL syndrome scale score and the same syndrome scale score at the next timepoint, were significant and positive. This indicates that higher scores on one scale significantly predict higher scores on the same scale at the next timepoint when controlling for the effects of all seven other scales. Centrality estimates for each scale are represented in Fig. 2. Outstrength centrality was highest for the thought problems scale, while social and anxious/depressed problems demonstrated the greatest instrength centrality. Withdrawn/depressed problems and rule-breaking behaviors showed the greatest bridge in-degree centrality and bridge out-degree centrality, demonstrating that they accounted for the strongest predictive relationships between internalizing and externalizing problem communities.

**Table 1:** Temporal Network Parameters and Inclusions Probabilities for Total Sample

From	To	*β*	*p*	Inclusion %	Type
Anxious/Depressed	Anxious/Depressed	0.091	< 0.001	92.0%	pos
Anxious/Depressed	Somatic	0.049	< 0.001	59.2%	pos
Anxious/Depressed	Social	0.089	< 0.001	89.9%	pos
Anxious/Depressed	Thought	0.066	< 0.001	81.8%	pos
Withdrawn/Depressed	Anxious/Depressed	-	-	66.1%	neg
Withdrawn/Depressed	Withdrawn/Depressed	0.104	< 0.001	91.5%	pos
Withdrawn/Depressed	Rule Breaking	0.067	< 0.001	64.5%	pos
Somatic	Somatic	0.116	< 0.001	97.4%	pos
Somatic	Social	-0.042	0.003	49.4%	neg
Social	Anxious/Depressed	0.067	< 0.001	87.7%	pos
Social	Social	0.158	< 0.001	99.9%	pos
Social	Rule Breaking	0.047	< 0.001	77.3%	pos
Thought	Anxious/Depressed	0.032	0.006	88.8%	pos
Thought	Somatic	-	-	77.2%	pos
Thought	Thought	0.099	< 0.001	99.0%	pos
Attention	Attention	0.074	< 0.001	92.9%	pos
Rule Breaking	Withdrawn/Depressed	0.033	< 0.001	52.6%	pos
Rule Breaking	Rule Breaking	0.109	< 0.001	99.3%	pos
Aggressive	Withdrawn/Depressed	0.035	0.005	42.3%	pos
Aggressive	Social	-	-	51.9%	neg
Aggressive	Thought	0.049	< 0.001	53.9%	pos
Aggressive	Attention	0.042	0.002	51.8%	pos
Aggressive	Aggressive	0.166	< 0.001	100%	pos

Standardized beta coefficients (*β*), p-values (*p*), bootstrapped inclusion probabilities (inclusion %), and type of relationship (positive = pos; negative = neg) of the predictive effects of each symptom domain (from) on each other (to) at the next timepoint. Only significant (*p* < 0.05) and/or robust (inclusion probability > 50%) effects are shown (“- “ indicates that the effect was not estimated in the final model after applying the model search procedure)

The robustness of the final model to sampling variation was examined by applying the same model search procedure to 1,000 bootstrapped samples and calculating the number of times each edge was included in the final model. All robust autoregressive effects and edges that were included in more than 50% of the models are shown in Fig. 1b. Inclusion probabilities of all robust autoregressive effects and edges are reported in Table 1. Of the 20 significant edges and autoregressive effects in the temporal network from the final model derived from the original sample, 18 were found to be robust in the models derived from the 1,000 bootstrapped samples. Two significant edges were not robust, representing potential false positives. Additionally, the Panel GVAR models of the bootstrapped samples produced three edges that were robust, but they were not included in the final model derived from the original sample, representing potential false negatives.

### Gender-Specific Network Structure

Two separate temporal networks of the eight CBCL syndrome scales were estimated for participants who identified as female (*n* = 3,035) and male (*n* = 3,366). The temporal network for girls is shown in Fig. 1a, and all significant edges included in the final model are shown in Table 2. The final model for girls demonstrated good fit (CFI = 0.988, TLI = 0.985, RMSEA = 0.0309). All autoregressive effects were significant and positive. Centrality estimates for each scale included in the temporal network for girls are represented in Fig. 2. Outstrength centrality was highest for thought problems, while instrength centrality was highest for the anxious/depressed scale. Anxious/depressed problems showed the greatest bridge in-degree centrality, while aggressive behaviors demonstrated the greatest bridge out-degree centrality.

**Table 2:** Temporal Network Parameters for Female and Male Participants

From	To	*β* (Female)	*p* (Female)	*β* (Male)	*p* (Male)	Δχ^2^(1)	*p*
AnxDep	AnxDep	0.147	< 0.001	-	-	1.34	0.246
AnxDep	Somatic	0.059	0.002	-	-	0.30	0.585
AnxDep	Social	0.076	0.004	-	-	0.17	0.677
AnxDep	Thought	0.100	< 0.001	-	-	0.06	0.807
AnxDep	Attention	-	-	-0.072	0.002	3.34	0.068
WithDep	AnxDep	-0.051	.002	-	-	0.11	0.740
WithDep	WithDep	0.066	.005	0.127	< 0.001	0.51	0.475
WithDep	RuleBreak	0.048	.011	0.073	< 0.001	0.02	0.884
Somatic	Somatic	0.138	< 0.001	0.109	< 0.001	1.50	0.221
Somatic	Social	-	-	-0.065	0.001	6.26	0.012
Social	AnxDep	0.056	0.003	0.057	< 0.001	0.79	0.373
Social	Social	0.107	< 0.001	0.202	< 0.001	0.86	0.355
Social	RuleBreak	0.048	0.004	0.056	0.001	0.07	0.791
Thought	AnxDep	0.081	< 0001	0.030	0.031	0.57	.450
Thought	WithDep	0.041	0.023	-	-	3.21	.073
Thought	Social	0.040	0.041	-	-	1.50	0.221
Thought	Thought	0.111	< 0.001	0.136	< 0.001	0.35	0.555
Thought	Attention	-	-	0.058	0.010	8.22	< 0.001
Attention	Social	0.040	0.003	-	-	1.40	0.237
Attention	Attention	0.088	< 0.001	0.069	0.004	0.01	.937
RuleBreak	AnxDep	-0.034	0.026	0.095	< 0.001	17.00	< 0.001
RuleBreak	WithDep	-	-	0.100	< 0.001	11.60	< 0.001
RuleBreak	Somatic	-	-	0.067	< 0.001	6.35	0.012
RuleBreak	Social	-	-	0.082	< 0.001	11.24	0.001
RuleBreak	Thought	-0.052	0.003	0.069	0.001	19.34	< 0.001
RuleBreak	RuleBreak	0.080	0.001	0.172	< 0.001	5.04	0.025
RuleBreak	Aggressive	-	-	0.060	0.005	2.07	0.150
Aggressive	AnxDep	-0.048	0.001	-	-	4.72	0.030
Aggressive	Somatic	-0.058	< 0.001	-	-	13.76	< 0.001
Aggressive	Social	-0.059	0.001	-	-	1.14	0.285
Aggressive	Thought	-	-	0.045	0.003	2.86	0.091
Aggressive	Attention	-	-	0.072	< 0.001	6.32	0.012
Aggressive	Aggressive	0.179	< 0.0001	0.088	< 0.001	2.60	0.106

Standardized beta coefficients (*β*) and *p*-values (*p*) of the predictive effects of each symptom domain (from) on each other (to) at the next timepoint for female and male participants. Only effects that were significant for female and/or male participants are shown (“- “ indicates that the effect was not estimated in the final model after applying the model search procedure). Chi-square difference (Δχ^2^(1)) in model fit when constraining each individual effect across genders is also reported with corresponding *p-*value. The symptom domains are: Anxious/Depressed Problems (AnxDep), Withdrawn/Depressed Problems (WithDep), Somatic Complaints (Somatic), Social Problems (Social), Thought Problems (Thought), Attention Problems (Attention), Rule-Breaking Behavior (RuleBreak), and Aggressive Behavior (Aggressive)

The temporal network for boys is shown in Fig. 1a, and all significant edges included in the final model are shown in Table 2. The final model for boys demonstrated good fit (CFI = 0.988, TLI = 0.986, RMSEA = 0.0315). All autoregressive effects were significant and positive, with the exception of the autoregressive effect of anxious/depressed problems on itself at the next timepoint, which was not significant. Centrality estimates for each scale included in the temporal network for boys are represented in Fig. 2. Outstrength centrality was highest for rule-breaking behaviors, while attention problems demonstrated the lowest outstrength centrality with no significantly predictive relationships on other scales at later timepoints. Somatic problems demonstrated the highest instrength centrality. Internalizing symptom clusters showed overall greater bridge in-degree centrality than externalizing symptom clusters, while rule-breaking behaviors demonstrated the highest out-degree bridge centrality.

Robustness of the final models estimated from female and male participants were examined using the same bootstrapping strategy described above. All robust autoregressive effects and edges that were included in more than 50% of the models for girls and boys are shown in Fig. 1b. Inclusion probabilities of all autoregressive effects and edges that were robust in the female and/or male temporal networks are reported in Table S3. Of the 24 significant edges and autoregressive effects in the temporal network from the final model derived from the female participants, 21 were robust in the models derived from the 1,000 bootstrapped samples. Three significant edges were not robust, representing potential false positives. Additionally, bootstrapped models produced two edges that were robust but not included in the final model derived from the original female sample, representing potential false negatives.

Of the 22 significant edges and autoregressive effects in the temporal network from the final model derived from the male participants, 21 were robust in the models derived from the 1,000 bootstrapped samples. One edge was not robust, representing a potential false positive finding. Additionally, bootstrapped models produced one autoregressive effect and six edges that were robust but not included in the final model derived from the original male sample, representing potential false negatives.

### Gender Difference Findings

Of the 35 total autoregressive effects and edges that were robust in either the bootstrapped female or male temporal networks, only eight autoregressive effects and nine edges were robust for both girls and boys (see Table S3). Additionally, three edges that were robust for both girls and boys differed in directionality (i.e., positive or negative) between boys and girls, specifically the predictive relationships of rule-breaking behavior on anxious/depressed problems, rule-breaking behavior on thought problems, and aggressive behaviors on somatic complaints.

When estimating the Panel GVAR model as a multi-group model, constraining all auto-regressive effects and edges to be equal across gender groups led to a significant drop in model fit (Δχ^2^(64) = 139.97 [p < 0.0001]), indicating that temporal network structure is unlikely to be equivalent overall. One auto-regressive effect and 10 edges were found to lead to a significant drop in model fit when individually constrained to be equal across gender groups (see Table 2). Notably, predictive relationships of externalizing problems (i.e., rule-breaking and aggressive behaviors) with other symptom clusters were unlikely to be equivalent across groups. Further examination of these edges across genders shows that, while externalizing problems tended to negatively predict other symptom cluster for girls, externalizing problems positively predicted other symptom clusters for boys (see Table 2). This is also apparent in comparing bridge out-degree centrality for rule-breaking behavior in which the magnitude of predictive relationships of rule-breaking behaviors on internalizing symptoms is substantially higher for boys than girls (see Fig. 2c).

## Discussion

Childhood is a period of vulnerability for the development of psychopathology that frequently extends into adulthood. Heterogenous symptom presentation and widespread disorder co-occurrence, including between internalizing and externalizing disorders, contradicts conventional views that psychological disorders are discrete entities. Delineating symptom trajectories from a transdiagnostic approach may resolve disorder heterogeneity and co-occurrence, yielding potential targets for intervention. The present study tested longitudinal relationships and potential gender differences among eight CBCL transdiagnostic symptom clusters in 6,414 ABCD study participants over three timepoints across 2 years using Panel GVAR models. Results demonstrated numerous significant predictive relationships, both positive and negative, among the symptom clusters, allowing inferences to be made about the centrality of individual symptoms clusters and the identification of potential symptom clusters that serve as a bridge between internalizing and externalizing symptoms. Specifically, rule-breaking behaviors, aggressive behaviors, and withdrawn/depressed problems emerged as important symptom domains in the development of co-occurring internalizing and externalizing disorders, though patterns differed between female and male participants, suggesting divergent developmental pathways of psychopathology.

### Symptom Centrality

The present study shows that outstrength centrality is relatively high for thought problems. These results differ from past findings that depression and worry are most predictive of other symptoms at future timepoints (Funkhouser et al., 2021). Funkhouser et al. (2021) as well as Speyer et al. (2022) found attention problems to be highly predictive of other symptoms at future timepoints, a finding that was not replicated in the present study, with attention problems having the lowest outstrength centrality. Interestingly, Speyer et al. (2022) theorized that behavioral difficulties in ADHD, specifically hyperactivity and inattention, may lead to increased struggle with peer interactions and exclusion by peers, potentially leading to antisocial behavior and conduct problems. Our results provide evidence of effects in the opposite direction, with aggressive problems positively predicting later attention problems, particularly for boys. Importantly, Speyer et al. (2022) analyzed children with a broader age range, and it is possible that the results of the current study are indicative of a pattern unique to the age range of the current sample, nine to twelve year-olds.

### Bridge Symptoms

Bridge symptoms in our temporal networks are represented by symptom clusters that show predictive relationships between internalizing and externalizing problems. Previous literature investigating cascade models has yielded mixed results with some studies showing bidirectional relationships between internalizing and externalizing problems (e.g., Achenbach & Rescorla, 2000; Mesman et al., 2001) and some work showing more restricted unidirectional effects of externalizing problems on future internalizing problem (e.g., Masten & Cicchetti, 2010; D’urso & Symonds, 2022). Results of the present study are generally supportive of a more unidirectional effect; externalizing problem symptom clusters had overall greater bridge out-degree centrality than internalizing problem symptom clusters, while internalizing problems had greater bridge in-degree centrality than externalizing problems. However, a significant and robust positive predictive relationship of withdrawn/depressed problems on rule-breaking behaviors at later timepoints was also found. Additionally, these findings are consistent with past research that has found depression to be a bridge symptom between internalizing and externalizing disorders (McElroy et al., 2018a, b).

### Gender Differences

Several differences in the patterns of predictive relationships between symptom clusters across female and male participants were noted. For symptom centrality, anxious/depressed problems showed the highest outstrength centrality for girls while showing low outstrength centrality for boys. Additionally, rule-breaking behaviors showed the highest outstrength centrality for boys but relatively lower outstrength centrality for girls. Another key difference was that externalizing problems negatively predicted other symptom clusters at later timepoints for girls, while externalizing problems positively predicted other symptom clusters at later timepoints for boys. The finding of a negative longitudinal relationship between externalizing problems and other symptom domains in girls runs contrary to the adjustment erosion hypothesis (Moilanen et al., 2010), which theorizes that early externalizing problems interfere with academic progress and peer relations leading to development of internalizing problems. Interestingly, Panayiotou and Humphrey (2018) found a similar pattern of initial externalizing problems predicting less internalizing problems later on, though this pattern was true for both boys and girls. They hypothesized that engagement in externalizing behaviors, particularly with delinquent peers, may lead to greater self-esteem by alleviating internalizing symptoms or that externalizing behaviors may trigger more attention from teachers which acts as a protective factor. It is possible that these hypothesized mechanisms may function differently between male and female identifying participants in the present study, leading to differences in effects of early externalizing behaviors. For example, rule-breaking behaviors positively predicted later social problems, specifically for boys but not for girls in our sample, and social problems positively predicted later internalizing problems. Therefore, male identifying participants in our sample showed a pattern consistent with the adjustment erosion hypothesis: early externalizing problems, specifically rule-breaking behaviors, predicted greater social problems, including problems with peer relations, that in turn positively predicted later internalizing problems, specifically anxious/depressed problems. In contrast, female identifying participants showed an opposite pattern from what would be expected by the adjustment erosion hypothesis: early externalizing problems, specifically aggressive and rule-breaking behaviors, predicted fewer future social and internalizing problems. This suggests that the mechanisms hypothesized by Panayiotou and Humphrey (2018) are more influential for girls rather than boys, leading to observed differences in effects of early externalizing behaviors, though factors such as self-esteem and attention from teachers were not measured, limiting conclusions.

Additionally, within externalizing problems, out-degree bridge centrality of aggressive behaviors was greater than rule-breaking behaviors for girls, but the opposite was true for boys. Additionally, internalizing problems, specifically withdrawn/depressed problems, positively predicted future externalizing problems, specifically rule-breaking behaviors in both female and male participants. However, the positive predictive relationship of withdrawn/depressed problems on rule-breaking behaviors was not robust for female participants. Taken together, these results indicate that developmental cascades between internalizing and externalizing problems may be more bidirectional for boys but more unidirectional for girls. Several explanations exist for mechanisms through which externalizing behaviors can lead to internalizing problems. For example, the irritable depression model hypothesizes that depressed mood can manifest as irritability which leads to conduct problems over time (Wolff & Ollendick, 2006). Interestingly, there is some evidence that irritability presents more commonly as a symptom of depression in both adult men and boys compared to women and girls (Khesht-Masjedi et al., 2017; Winkler et al., 2006). This suggests that the mechanistic pathway proposed by the irritable depression model may be more applicable to boys compared to girls, potentially explaining the presence of a robust positive longitudinal effect of internalizing problems on future externalizing problems in boys, but not in girls, in our sample.

### Limitations and Future Directions

Though Panel GVAR models can accurately describe predictive relationships in panel data with as little as three timepoints (see Epskamp, 2020), the inclusion of additional timepoints beyond the 2-year duration of the current study is necessary to test the consistency of the patterns of predictive relationships found in the present study. Similarly, the low number of measurement occasions may explain the suboptimal stability of centrality estimates when applying case-drop subset bootstrapping. Additionally, there is evidence that patterns of developmental cascades change during development (Moilanen et al., 2010), and GVAR models’ assumptions of equal relations over time limit ability to detect changes in developmental patterns. Therefore, though GVAR models in this study found effects between symptom clusters that were consistent across all three timepoints, these effects may not be generalizable beyond the age group considered by this study. As the ABCD study continues gathering more data and participants continue developing, it will be important to examine whether patterns of relationships among symptom clusters change during different stages of preadolescence and adolescence. Additionally, although the focus on the transdiagnostic symptom clusters represented by the CBCL syndrome scales allows for conclusions about broad patterns in development of psychopathology, the lack of granularity in terms of specific symptoms limits conclusions about which specific symptoms within a given symptom cluster serve as central symptoms or bridge symptoms. The use of parent-report on the CBCL is also a limitation as parent-report may not reflect participants’ symptomatology, particularly given low parent–child concordance on internalizing symptoms (Rey et al., 1992). Finally, past reported psychopathology and gender effects are only two contributors to psychopathology trajectories, and as past literature has stated, consideration of how protective and risk factors also contribute to the development of psychopathology in youth is necessary (Fried et al., 2017; Goh & Martel, 2021). Possible protective and risk factors include school engagement and prosocial behaviors as well as abuse or neglect.

### Constraints on Generality

Though recruitment for the ABCD study was intended to be nationally representative, there are certain limitations to the generalizability of this sample to the general U.S. population (Heeringa & Berglund, 2020). Notably, when compared to the American Community Survey (ACS) conducted by the U.S. Bureau of Census, 40.7% of the ABCD sub-sample included in the present study had an annual combined family income greater than $100,000 compared to 17.3% in the ACS. As previously reported, the ABCD sample also underrepresents children attending schools in rural settings (Heeringa & Berglund, 2020). Given these limitations, caution should be exercised in generalizing these findings to the broader U.S. population.

### Clinical Implications

Symptom clusters with high outstrength centrality and bridge out-degree centrality can represent causal mechanisms in the development of psychopathology in youth (Borsboom & Cramer, 2013; Cramer et al., 2010). Therefore, focused assessment of these symptom clusters can help identify youth who are at risk for developing future psychopathology, and treatment of these symptom clusters may prevent development of future comorbid problems. Based on the total sample panel GVAR model, anxious/depressed problems and aggressive behaviors represent highly influential symptom clusters, while withdrawn/depressed problems and rule-breaking behavior represent bridge symptoms between externalizing and internalizing problems. Early screening and intervention for these symptom clusters may be important for prevention of future psychopathology. However, female participants demonstrated a different pattern, where anxious/depressed problems and aggressive behavior were still influential symptom clusters, but aggressive behavior negatively predicted future increases in other symptom clusters. Additionally, only withdrawn/depressed problems represented a bridge symptom between externalizing and internalizing problems for female participants. Male participants also demonstrated a different pattern, where rule-breaking behaviors were the influential symptom cluster rather than anxious/depressed problems and aggressive behavior. However, rule-breaking behavior and withdrawn/depressed problems represented bridge symptoms between externalizing and internalizing problems for male participants. Our results imply that screening and early interventions targeting anxious/depressed problems and withdrawn/depressed problems for girls, and rule-breaking behavior and withdrawn/depressed problems in boys, on average, could have broad effects in the reduction and/or prevention of psychopathology. Lastly, the symptom trajectories yielded here, and associated gender differences may provide promising pathways for understanding disorder (dis)continuity and co-occurrence, although longer-term longitudinal data are needed to test pathway stability and divergence across development. Still, caution should be exercised to avoid over-interpretation of these results. Present results only reflect within-person temporal relationships for the average participant, and individualized within-person longitudinal modeling will yield more precise information on symptom centrality and causal structure that is better suited for the treatment of an individual patient (Epskamp et al., 2018; Goh & Martel, 2021).

## Conclusion

The present study tested the temporal relationships among psychopathology symptom domains in a homogeneous age sample of youth (from the ABCD study) across three timepoints over two years. Anxious/depressed problems and aggressive behaviors emerged as the two symptom clusters that were most predictive of increases in other symptom clusters at later timepoints. Additionally, rule-breaking behaviors, aggressive behaviors, and withdrawn/depressed problems demonstrated evidence of bridge symptoms between externalizing and internalizing problems, and there were bi-directional predictive relationships between externalizing and internalizing problems. Notably, patterns of symptom centrality and bridge symptoms between externalizing and internalizing disorders differed between boys and girls. Present results may inform screening and intervention strategies for youth at-risk for psychopathology development. Future studies incorporating risk and protective factors as well as consideration of specific symptoms within symptom domains will refine developmental trajectories.

### Supplementary Information

Below is the link to the electronic supplementary material.

Supplementary file1 (DOCX 28 KB)

## Data Availability

Data used in the preparation of this article were obtained from the Adolescent Brain Cognitive Development (ABCD) Study (https://abcdstudy.org), held in the NIMH Data Archive (NDA). This is a multisite, longitudinal study designed to recruit more than 10,000 children age 9–10 and follow them over 10 years into early adulthood. The ABCD Study^®^ is supported by the National Institutes of Health and additional federal partners under award numbers U01DA041048, U01DA050989, U01DA051016, U01DA041022, U01DA051018, U01DA051037, U01DA050987, U01DA041174, U01DA041106, U01DA041117, U01DA041028, U01DA041134, U01DA050988, U01DA051039, U01DA041156, U01DA041025, U01DA041120, U01DA051038, U01DA041148, U01DA041093, U01DA041089, U24DA041123, U24DA041147. A full list of supporters is available at https://abcdstudy.org/federal-partners.html. A listing of participating sites and a complete listing of the study investigators can be found at https://abcdstudy.org/consortium_members/. ABCD consortium investigators designed and implemented the study and/or provided data but did not necessarily participate in analysis or writing of this report. This manuscript reflects the views of the authors and may not reflect the opinions or views of the NIH or ABCD consortium investigators. The ABCD data repository grows and changes over time. The ABCD data used in this report came from https://doi.org/10.15154/1520926. DOIs can be found at NDAR-https://doi.org/10.15154/1520926.
