# Supplementary material for: Developmental Trajectories of Internalizing and Externalizing Symptoms in Youth and Associated Gender Differences: A Directed Network Perspective
Source: Res Child Adolesc Psychopathol. 2023 Aug 7;51(11):1627–39. doi: 10.1007/s10802-023-01106-4 (PMC10627904; doi:10.1007/s10802-023-01106-4)
Supplement: Supplementary file 1 — Supplementary file1 (DOCX 28 KB) [file 10802_2023_1106_MOESM1_ESM.docx]

**Developmental Trajectories of Internalizing and Externalizing Symptoms in Youth and Associated Gender Differences: A Directed Network Perspective**

**Supplementary Materials**

**Table S1**

*Demographic Information for Sample*

| Demographic | n (%) |
| --- | --- |
| Gender identity |  |
| Male | 3366 (52.5%) |
| Female | 3035 (47.3%) |
| Trans male | 1 (0.02%) |
| Trans female | 3 (0.05%) |
| Gender queer  Different Identity | 1 (0.02%) |
| Different identity | 3 (0.05%) |
| Refused to answer | 4 (0.06%) |
| Don’t know | 1 (0.02%) |
|  |  |
| Race |  |
| White | 5039 (78.6%) |
| Black/African American | 1088 (17.0%) |
| American Indian/Native American | 226 (3.5%) |
| Alaska Native | 2 (0.03%) |
| Native Hawaiian | 15 (0.2%) |
| Samoan | 4 (0.06%) |
| Other Pacific Islander | 25 (0.4%) |
| Asian Indian | 47 (0.7%) |
| Chinese | 123 (1.9%) |
| Filipino/a | 92 (1.4%) |
| Japanese | 44 (0.7%) |
| Korean | 58 (0.9%) |
| Vietnamese | 33 (0.5%) |
| Other Asian | 42 (0.7%) |
| Other Race | 364 (5.7%) |
| Refuse to answer/Don't know | 77 (1.2%) |
| Ethnicity |  |
| Hispanic/Latino | 1220 (19.0%) |
| Not Hispanic/Latino | 5110 (79.7%) |
| Refuse to answer/Don't know | 84 (1.3%) |
| Current grade |  |
| 1st Grade | 1 (0.01%) |
| 2nd Grade | 24 (0.3%) |
| 3rd Grade | 913 (14.2%) |
| 4th Grade | 2898 (45.2%) |
| 5th Grade | 2389 (37.2%) |
| 6th Grade | 187 (2.9%) |
| 7th Grade | 2 (.03%) |
| Combined family income |  |
| Less than $5,000 | 151 (2.4%) |
| $,5000 to $,11999 | 186 (2.9%) |
| $12,000 to $15,999 | 124 (1.9%) |
| $16,000 to $24,999 | 248 (3.9%) |
| $25,000 to $34,999 | 348 (5.4%) |
| $35,000 to $49,999 | 518 (8.1%) |
| $50,000 to $74,999 | 836 (13.0%) |
| $75,000 to $99,999 | 932 (14.5%) |
| $100,000 to $199,999 | 1891 (29.5%) |
| $200,000 and greater | 718 (11.2%) |
| Refused/Don't know | 462 (7.2%) |

*Note.* Parent-reported gender identity, race, ethnicity, current grade, and combined family income information at 2-year follow-up.

**Table S2**

*Descriptive Statistics for Child Behavior Checklist Subscales*

|  |  | Anxious/ Depressed | Withdrawn/ Depressed | Somatic Complaints | Social Problems | Thought Problems | Attention Problems | Rule-Breaking Behavior | Aggressive Behavior |
| --- | --- | --- | --- | --- | --- | --- | --- | --- | --- |
| Baseline | Mean | 53.48 | 53.51 | 54.88 | 52.79 | 53.79 | 53.9 | 52.78 | 52.83 |
|  | *SD* | 5.96 | 5.79 | 6.05 | 4.74 | 5.9 | 6.17 | 4.9 | 5.53 |
|  | Skew | 2.22 | 5.16 | 1.32 | 2.6 | 1.93 | 2.47 | 2.35 | 2.76 |
|  | Kurtosis | 5.48 | 5.26 | 1.23 | 8.24 | 3.25 | 7.62 | 5.51 | 9.04 |
| 1-Year Follow-up | Mean | 53.52 | 53.73 | 54.74 | 52.58 | 53.79 | 53.71 | 52.56 | 52.58 |
|  | *SD* | 5.97 | 5.93 | 6.01 | 4.55 | 5.96 | 5.98 | 4.73 | 5.29 |
|  | Skew | 2.19 | 2.08 | 1.39 | 2.7 | 1.94 | 2.47 | 2.49 | 2.91 |
|  | Kurtosis | 5.13 | 4.79 | 1.46 | 8.75 | 3.24 | 7.4 | 6.28 | 9.95 |
| 2-Year Follow-up | Mean | 53.22 | 53.46 | 54.61 | 52.58 | 53.63 | 53.46 | 52.09 | 52.37 |
|  | *SD* | 5.8 | 5.74 | 5.9 | 4.76 | 5.71 | 5.56 | 4.12 | 4.92 |
|  | Skew | 2.4 | 2.39 | 1.5 | 2.57 | 1.92 | 2.45 | 2.81 | 3.03 |
|  | Kurtosis | 6.48 | 7.26 | 2.04 | 7.47 | 3.31 | 7.7 | 8.81 | 11.31 |

*Note.* Mean, standard deviation (*SD*), skew, and kurtosis values for the eight Child Behavior Checklist subscales: Anxious/Depressed Problems, Withdrawn/Depressed Problems, Somatic Complaints, Social Problems, Thought Problems, Attention Problems, Rule-Breaking Behavior, and Aggressive Behavior.

**Table S3**

*Bootstrapped Inclusion Probabilities for Temporal Network Parameters for Females and Males*

| From | To | Female Inclusion % | Female Type | Male Inclusion % | Male Type |
| --- | --- | --- | --- | --- | --- |
| AnxDep | AnxDep | 90.0% | pos | 62.9% | pos |
| AnxDep | Somatic | 54.5% | pos | 37.9% | pos |
| AnxDep | Social | 61.9% | pos | 70.0% | pos |
| AnxDep | Thought | 65.9% | pos | 57.4% | pos |
| WithDep | AnxDep | 67.3% | neg | 35.9% | neg |
| WithDep | WithDep | 54.9% | pos | 89.2% | pos |
| WithDep | Somatic | 20.6% | neg | 53.0% | neg |
| WithDep | RuleBreak | 47.3% | pos | 54.3% | pos |
| WithDep | Aggressive | 8.9% | pos | 52.1% | neg |
| Somatic | Somatic | 92.9% | pos | 73.1% | pos |
| Somatic | Social | 16.7% | pos | 77.3% | neg |
| Social | AnxDep | 72.4% | pos | 58.1% | pos |
| Social | Social | 88.7% | pos | 99.6% | pos |
| Social | RuleBreak | 58.8% | pos | 54.3% | pos |
| Thought | AnxDep | 77.3% | pos | 62.1% | pos |
| Thought | WithDep | 69.7% | pos | 9.8% | pos |
| Thought | Somatic | 52.8% | pos | 60.3% | pos |
| Thought | Social | 52.8% | pos | 13.7% | pos |
| Thought | Thought | 81.2% | pos | 97.2% | pos |
| Thought | Attention | 25.9% | neg | 78.0% | pos |
| Attention | Attention | 70.4% | pos | 66.3% | pos |
| RuleBreak | AnxDep | 58.7% | neg | 78.8% | pos |
| RuleBreak | WithDep | 25.4% | neg | 85.9% | pos |
| RuleBreak | Somatic | 16.8% | neg | 65.6% | pos |
| RuleBreak | Social | 30.0% | neg | 74.9% | pos |
| RuleBreak | Thought | 80.3% | neg | 66.0% | pos |
| RuleBreak | RuleBreak | 63.0% | pos | 99.3% | pos |
| RuleBreak | Aggressive | 6.9% | neg | 54.6% | pos |
| Aggressive | WithDep | 51.2% | pos | 13.7% | pos |
| Aggressive | Thought | 13.0% | neg | 62.6% | pos |
| Aggressive | Somatic | 85.3% | neg | 51.3% | pos |
| Aggressive | Social | 52.8% | neg | 25.6% | neg |
| Aggressive | Attention | 16.0% | neg | 78.1% | pos |
| Aggressive | Aggressive | 99.8% | pos | 96.9% | pos |

*Note.* Inclusion probability (inclusion %) and type of relationship (positive = pos; negative = neg) of each robust (inclusion probability > 50%) predictive effect of each symptom domain (from) on each other (to) at the next timepoint for the female and male participant groups. The symptom domains are: Anxious/Depressed Problems (AnxDep), Withdrawn/Depressed Problems (WithDep), Somatic Complaints (Somatic), Social Problems (Social), Thought Problems (Thought), Attention Problems (Attention), Rule-Breaking Behavior (RuleBreak), and Aggressive Behavior (Aggressive).
